# Transperitoneal laparoscopic adrenalectomy for metachronous contralateral adrenal metastasis from renal cell carcinoma: a case report

**DOI:** 10.1186/1757-1626-1-185

**Published:** 2008-09-26

**Authors:** Evangelos Zacharakis, Maria J Ribal, Emmanouil Zacharakis, Hiten RH Patel

**Affiliations:** 1 Section of Laparoscopic Urology, The Institute of Urology, University College Hospital, London, UK; 2 Academic Surgical Unit, St Mary's Hospital, London, UK

## Abstract

**Background:**

We report a case of metachronous solitary metastasis of renal cell carcinoma to the contralateral adrenal gland treated by laparoscopic transperitoneal adrenalectomy.

**Case presentation:**

A 58-year-old man presented to our institution for regular follow up, 2 years after a right radical nephrectomy with preservation of the ipsilateral adrenal gland, for a primary renal cell carcinoma. The patient remained asymptomatic but an abdominal computed tomography scan on follow up revealed a 6.5 × 4 cm^2 ^mass in the left adrenal gland. A positron emission tomography scan was also performed to rule out other possible metastases, and a magnetic resonance imaging scan was used for accurate localization and determination of resectability of the adrenal tumour. A bone scan, metabolic screen, liver and renal function tests were all within normal limits. A laparoscopic transperitoneal adrenalectomy was then performed. The postoperative period was uneventful, and the patient was discharged on postoperative day two. The patient remains in satisfactory condition and no recurrence or adrenal insufficiency has been observed during 12 months follow up.

**Conclusion:**

Metachronous contra lateral adrenal metastases from primary renal cell carcinoma are very rare but should always be suspected in any nephrectomised patient presenting with an adrenal tumour. Regular follow up in these patients accompanied with computed tomography imaging may help the surgeon to detect early lesions. Laparoscopic transperitoneal adrenalectomy is feasible, safe and effective, with minimal trauma to the patient.

## Background

Renal cell carcinoma (RCC) may present with distant metastasis at the time of diagnosis. It has been estimated that at the time of diagnosis 20% to 50% of patients are beyond cure, and less than 5% of those with metastatic disease are alive at 5 years [1]. However, it has been reported that improved survival can be achieved when solitary metastases are excised together with the primary RCC [2]. Adrenal metastases from RCC are frequently found at autopsy [3]. These lesions tend to be detected synchronously with the renal tumour, and only a few are detected metachronously on follow up after nephrectomy [3].

In recent years, the laparoscopic approach has been reported to offer the well-known benefits of minimally invasive surgery over the traditional open technique for adrenal tumours, and can be performed either transperitoneally or retroperitoneally [4]. The transperitoneal approach is reserved for malignant adrenal disease as it has the benefit of a wider working space and readily identifiable anatomical landmarks [5]. It might be assumed that the benefits of the laparoscopic approach for primary adrenal tumours could be applied for metastatic adrenal disease as well. We report a case of laparoscopic transperitoneal adrenalectomy in a patient with metachronous solitary metastasis of RCC to the contra lateral adrenal gland.

## Case presentation

**Figure 1:**
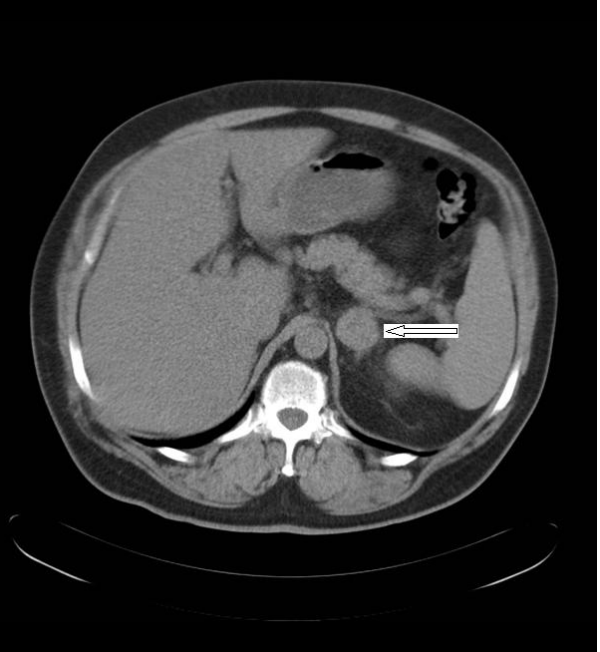
CT scan showing an adrenal metastasis to the contralateral gland, 2 years after a right nephrectomy for primary RCC.

A 58-year-old man presented to our institution for regular follow up, 2 years after a right radical nephrectomy for a primary RCC. The patient remained asymptomatic but an abdominal computed tomography (CT) scan on follow up revealed a homogeneous 6.5 × 4 cm^2 ^mass in the left adrenal gland with no calcification (Figure 1). Furthermore, the positron emission tomography scan revealed a fluorodeoxyglucose (FDG)-avid enlarged left adrenal gland, with no evidence of FDG-avid para-aortic lymphadenopathy or any further abnormality of the right renal bed or adjacent areas. Radiological studies (abdominal and chest CT scan) before the radical nephrectomy had revealed a 12 cm heterogeneous soft tissue mass in mid pole in the right kidney consistent with an RCC. There was no involvement of the inferior vena cava or right renal vein and no evidence of distant metastases at that point. The patient then underwent a transperitoneal laparoscopic radical nephrectomy of the right kidney with preservation of the ipsilateral adrenal gland. The pathology report of the nephrectomy specimen had shown a Fuhrman grade 2 stage T2 (T2N0M0) RCC.

After the detection of the left adrenal tumour on follow up, the patient was admitted for further investigation and management. A MRI scan was used for accurate localization and determination of resectability of the adrenal tumour. A bone scan for metastatic evaluation was unremarkable. Laboratory tests, including full blood count, electrolytes, complete metabolic screen, liver and renal function were all within normal limits. In particular, metabolic evaluation included 24-hour urine collections for 17-ketosteroids, 17-hydrocorticoids, metanephrines, cortisol and vanillylmandelic acid, which were within normal limits. The American Society of Anesthesiologists (ASA) grade of the patient was III. Based on these findings and through a multidisciplinary team meeting, a laparoscopic transperitoneal adrenalectomy was considered for this patient.

After induction of general anaesthesia, a urinary catheter and a nasogastric tube were inserted. The patient was placed in the lateral decubitus position with the left side up. After creating the pneumoperitoneum, a 30° laparoscope was inserted and three additional working ports (5,5 and 10 mm) were placed under direct vision. A plane was established along the anterior surface of the left kidney, just lateral and dorsal to the spleen and tail of pancreas. This was accomplished by incising the splenorenal ligament and mobilizing the spleen laterally; the superior pole of the kidney and the adrenal gland with the tumour were thus exposed. The dissection along the anterior surface of the kidney and adrenal continued until the inferior pole and medial border of the adrenal was exposed. This allowed identification of the dissection plane between the anterior surface of the kidney and the inferior border of the adrenal. The dissection was continued along the inferior and medial aspect of the adrenal, staying close to the gland until the left adrenal vein was isolated and clipped. Final dissection and gland excision progressed from medial to lateral and inferior to superior. Before specimen extraction, the operative field was carefully inspected for haemostasis. Once haemostasis was ensured, the adrenal gland with the tumour was placed in a specimen retrieval sac and removed through a 10 mm trocar site. The operation was completed by releasing the pneumoperitoneum and closing the fascia of the 10 mm trocar site with absorbable sutures.

The histological examination of the specimen demonstrated a metastatic RCC with features similar to the primary renal tumour. All surgical margins were free of tumour. The specimen weighed 62 g and measured 6.5 × 4 × 3 cm^3^. The postoperative period was uneventful, and the patient was discharged on postoperative day two. The patient is well and has no recurrence or adrenal insufficiency 18 months post-surgery.

## Discussion

Approximately 25% of patients with RCC will already have multiple distant metastases at the time of presentation, including the lungs (55%), lymph nodes (38%), liver (35%) or bones (33%) [1]. Adrenal metastases from RCC are not uncommon, with autopsy studies showing an incidence of 6% to 23% [3]. However, in nephrectomised patients the incidence of solitary adrenal metastasis is 3% to the ipsilateral gland and only 0.7% to the contra lateral gland [6].

It has been suggested that the anatomic relationship between the kidney and the adrenal gland on the left side with common venous and lymphatic drainage predisposes towards adrenal gland metastases in left sided tumours [7]. With primary RCC it is believed that haematogenous metastasis to the adrenal gland is more common than direct extension, due to the rich blood supply of the adrenal gland and its high blood volume-to-unit weight ratio [7]. However, the underlying biological pathway for contralateral adrenal metastases (CAM) by RCC is still unknown [8]. Of interest, a published series of patients with metastatic spread to the contra lateral adrenal gland or kidney showed an 80% incidence of involvement of the renal vein or inferior vena cava by the primary tumour [9]. In our case, preoperative imaging of the RCC before radical nephrectomy had not revealed any involvement of the inferior vena cava or the right renal vein.

Adrenal metastases are usually anatomically and functionally silent, and patients rarely have symptoms or signs of adrenal insufficiency. Thus, if abdominal imaging is not used routinely during the follow up, an isolated adrenal metastasis from RCC could be missed during life [7]. A solitary metastasis to the contra lateral adrenal gland can cause confusion, particularly since the histological status maybe unclear [9]. The optimal diagnostic approach to a solitary contra lateral adrenal tumour in patients with a history of RCC is contentious and seems to differ from the management of 'incidentalomas' [8]. Radiological studies may facilitate the preoperative diagnosis but cannot determine with certainty whether an adrenal tumour in a patient with RCC is a primary adrenal neoplasm, an adrenal cortical adenoma or a metastasis [8]. The preoperative diagnosis of CAM begins with high suspicion based on a history of RCC. In these patients, the findings of a solitary adrenal tumour without elevated serum adrenocortical hormones are strongly suggestive of a metastatic lesion to the adrenal gland [7,9]. In our case, the normal metabolic screen and the recent history of RCC suggested adrenal metastasis as the most likely diagnosis.

The remote risk of developing CAM after primary radical nephrectomy supports the idea of sparing the adrenal gland in suitable patients who undergo radical nephrectomy [10]. Sparing the ipsilateral adrenal gland in radical nephrectomy would avoid the risk of adrenal insufficiency if the development of a tumour requires removal of the contra lateral adrenal gland, either at the time of nephrectomy or later [10]. The need for routine adrenalectomy during radical nephrectomy is questionable as the risk of an ipsilateral adrenal tumour developing after radical nephrectomy is low (3%). The only exception would be for tumours localized in the upper pole of the kidney and close to the adrenal gland [10].

Surgical removal is the only known effective treatment in patients with solitary adrenal metastasis, with 29% to 35% of them surviving 5 years or more [11]. Plawner showed that the 5-year survival of patients operated on for metachronous solitary RCC metastases to the contra lateral adrenal gland was lower than that for patients with synchronous adrenal metastases (20% and 40%, respectively) [11]. It has also been noted that patients diagnosed with adrenal metastasis a long time after nephrectomy had a better prognosis than those with a short interval to diagnosis [1]. Among patients who undergo nephrectomy and resection of solitary metastases, it has been reported that 30% have prolonged survival, many of them for more than 5 years. Therefore, aggressive treatment-excision of such lesions is indicated [2,11].

Because of the anatomical position of the adrenal gland and the small size of the lesions, laparoscopic adrenalectomy is an interesting approach to adrenal surgery. Studies comparing the laparoscopic and the open adrenalectomy reported statistically significant benefits in the laparoscopic group regarding the hospital stay, analgesic requirements, blood loss, return to normal activity and patient satisfaction [12]. Preliminary reports showed that these minimally invasive procedures may advantageously replace open surgery for removal of unilateral or bilateral tumours of the adrenal gland of less than 7 cm [5]. However, successful laparoscopic resections of tumours measuring up to 15 cm have also been reported [4].

There are four laparoscopic approaches to the adrenal gland, namely transperitoneal, lateral retroperitoneal, posterior retroperitoneal and transthoracic transdiaphragmatic. The transperitoneal approach was used first and is still the most popular technique as it provides a more ample working cavity, greater visibility and better instrument mobility [4]. The retroperitoneal approaches are preferred in patients with history of upper abdominal surgery and unilateral adrenal tumours less than 5 cm in diameter. Particularly in the case of adrenal cancer or pheochromocytoma, the transperitoneal adrenalectomy is considered the approach of choice, because of the wide exposure and as it allows inspection of extra-adrenal sites and 'en bloc' excision in case of extended disease [5]. In our case, the adrenal metastasis measured 6.4 × 4 cm^2 ^in preoperative imaging and was resected successfully through a laparoscopic transperitoneal approach, in a patient with a previous history of transperitoneal laparoscopic radical nephrectomy of the contra lateral kidney.

## Conclusion

Metachronous CAM from primary RCC are very rare but should always be suspected in any nephrectomised patient presenting with an adrenal tumour. Regular follow up in these patients with CT or MRI imaging may help the surgeon to detect these lesions early. Laparoscopic transperitoneal adrenalectomy seems to be a feasible, safe and effective approach for these rare cases.

## Abbreviations

ASA: American Society of Anesthesiologists; CAM: contralateral adrenal metastases; CT: computed tomography; FDG: fluorodeoxyglucose; RCC: renal cell carcinoma.

## Consent

Written informed consent was obtained from the patient for publication of this case report and any accompanying images. A copy of the written consent is available for review by the Editor-in-Chief of this journal.

## Competing interests

The authors declare that they have no competing interests.

## Authors' contributions

EvZ made substantial contributions to conception and design, acquisition and interpretation of data; has been involved in drafting the manuscript. MJR made substantial contributions to interpretation of data; has been involved in drafting the manuscript. EmZ made substantial contributions to analysis and interpretation of data and has been involved in revising the manuscript critically for important intellectual content. HRHP undertook the surgery and care of the patient as well as being involved in conception of the manuscript. He is senior author and guarantor for the manuscript. All authors read and approved the final manuscript.
